# Transcriptomic response to *Borrelia afzelii* infection in the skin of wild bank voles

**DOI:** 10.1128/spectrum.02574-25

**Published:** 2026-01-27

**Authors:** Joanna Różańska-Wróbel, Mateusz Konczal, Rocco F. Notarnicola, Jacek Radwan

**Affiliations:** 1 Evolutionary Biology Group, Institute of Environmental Biology, Faculty of Biology, Adam Mickiewicz University 236115 , Poznań, Poland; University of São Paulo, São Paulo, Brazil

**Keywords:** bank vole, *Borrelia afzelii*, Lyme disease, RNA-seq, WGCNA

## Abstract

Bank voles are one of the main reservoirs of tick-transmitted spirochete *Borrelia afzelii*, a causative agent of Lyme disease in humans in Europe. How the immune system deals with infection at the site of entry, that is, the skin, has not been explored in this species. Here, we used RNA sequencing to explore the transcriptomic response in the ear skin of wild bank voles infected with *B. afzelii*. We identified 54 differentially expressed genes, of which 37 showed upregulation, and 17 showed downregulation in infected voles compared to uninfected ones. Weighted gene co-expression network analysis identified five gene modules, which were positively or negatively correlated with infection status. Enrichment analysis revealed numerous biological processes and pathways related to immune response, extracellular matrix organization, metabolism, energy production, gene expression, and cell cycle regulation. Among immunity-related genes, pathways related to B-cell activity and antibody production were particularly upregulated. However, we found that the pro-inflammatory response is suppressed compared to that reported in humans, and we identified changes in the expression of genes related to the extracellular matrix, whose products are bound and colonized by *Borrelia*. These findings indicate a complex response of bank voles to *B. afzelii* infection and provide insight into the molecular processes associated with infection in a natural reservoir host.

**IMPORTANCE:**

Lyme disease is a common infectious disease in Europe and North America caused by *Borrelia burgdorferi sensu lato* spirochetes, which are transmitted through tick bites. While the infection can lead to severe symptoms in humans, including fatigue, fever, joint pain, and neurological disorders, natural reservoir hosts such as rodents typically remain asymptomatic, providing an important model for uncovering the molecular basis of infection tolerance. By comparing gene expression differences between *Borrelia*-infected and -uninfected individuals of a wild rodent species, the bank vole, we identified molecular pathways involved in the early response at the site of infection, the skin. Our findings revealed a reduced pro-inflammatory response, enhanced adaptive immune activation, particularly involving B-cell-mediated processes, and changes in extracellular matrix organization. These results provide insight into the immune strategy of reservoir hosts and may help explain why Lyme disease causes more severe symptoms in humans.

## INTRODUCTION

Infectious diseases are a major global health concern, affecting both humans and animals, with zoonotic pathogens being a key driver of emerging diseases (1–3). Many pathogens can infect multiple host species, although they can persist only in selected reservoir hosts (4). These natural reservoir hosts, crucial for the transmission and persistence of pathogens, often establish mild or asymptomatic infections. To understand the mechanisms that facilitate pathogen spread and survival, it is essential to study the coevolutionary dynamics of host-pathogen interactions in such systems and the molecular basis of host immune responses.

Lyme disease is one of the vector-borne zoonoses that has spread in the Northern Hemisphere over the last few decades (5). It is caused by spirochetes from the *Borrelia burgdorferi sensu lato* (s.l.) species complex, which are transferred to vertebrate hosts by *Ixodes* ticks (6). In humans, Lyme disease can develop with varying severity and symptoms, such as skin manifestations, carditis, arthritis, and neurological disorders (7). However, humans are only incidental hosts of *B. burgdorferi* s.l., whereas reservoir hosts include rodents and birds. In Europe, the primary causative agents of Lyme disease are the spirochetes *Borrelia afzelii* and *Borrelia garinii*, which exhibit host specialization. *B. afzelii* is primarily associated with rodents and *B. garinii* with birds (6). One of the main reservoir hosts of *B. afzelii* is the bank vole (*Clethrionomys glareolus*) (8, 9), a small rodent found in forests throughout Europe (10). *B. afzelii* infection in bank voles typically does not affect their body condition or survival (11), although it has been shown that infection can reduce their reproductive success (12). Considering the role of bank voles as a key reservoir for *B. afzelii*, examining their molecular response to infection is essential to understand the broader dynamics of the ecology of Lyme disease, as well as how the spirochete is maintained in natural ecosystems and adapts to the immune responses of hosts.

Here, we performed RNA sequencing (RNA-seq) to analyze the responses of the whole transcriptome to *B. afzelii* infection and identify candidate genes involved in host-pathogen interactions. While previous research investigated transcriptomic responses to *Borrelia* infection in bank voles (13) and other rodents (14–16), two of them focused on skin tissue (15, 16), which is the primary site of *Borrelia* infection and replication (17). However, these studies were conducted on *Peromyscus leucopus* and involved an experimental infection with *B. burgdorferi sensu stricto* (s.s.). Given its role as the primary site of infection, effective immune responses in the skin are key to controlling *Borrelia* infection and preventing its dissemination to other tissues. No investigation of skin expression patterns in response to *B. afzelii* has been carried out in bank voles. Here, we fill this gap by analyzing the whole transcriptome of 44 wild-caught voles, comparing those infected with *B. afzelii* to uninfected ones.

## MATERIALS AND METHODS

### Sample collection

Bank voles were sampled in August 2023 at three sites in Poland: Brok (52°42′01.0″N, 21°55′07.8″E), Długosiodło (52°45′49.6″N, 21°37′56.5″E), and Grobka (53°31′00.0″N, 20°37′28.9″E). All sites were forests with similar habitats characterized by dense undergrowth and alders (genus *Alnus*) as dominant trees. Bank voles were captured overnight in wooden live traps with a metal door, triggered by the animal stepping on a small platform inside. Grains and a piece of apple were used as bait in the traps. After capture, voles were weighed, anesthetized with isoflurane, and a ~3 mm ear biopsy was taken from each animal. All voles were released at the site of capture following sampling. The ear samples were then split; one half was stored in 1 mL of ethanol for DNA extraction, while the other was preserved in 1 ml of RNAlater Stabilization Solution (Thermo Fisher Scientific, Waltham, MA, USA) for RNA extraction. After 4–8 hours, RNAlater samples were frozen and stored at −20°C.

### DNA extraction, sex identification, and *Borrelia* screening

DNA was extracted from bank vole ear samples with the NucleoMag 96 Tissue Kit (Macherey-Nagel, Duren, Germany) and used to determine the sex and *Borrelia* infection status of the bank voles. Sex was identified using PCR with two primer sets. The first pair, P1-5EZ and P2-3EZ, amplified a 447 bp fragment of the ZFX/ZFY genes, which are present in both sexes (18), and served as a control for successful DNA amplification. The second pair, SRY-HMG-F and SRY-HMG-R, amplified a 202 bp fragment of the conserved HMG box region of the Sry gene, which is located on the Y chromosome and specific to males; the primers were originally designed based on the Sry gene sequence of *Mus musculus* (19). Duplex PCRs were performed using the Type-it Microsatellite PCR Kit (Qiagen, Hilden, Germany) according to the manufacturer’s protocol. Each reaction contained 1 µL of template DNA, 0.5 µL of each primer (final concentration: 0.2 µM), 12.5 µL of 2× Type-it Multiplex PCR Master Mix, and nuclease-free water up to 25 µL. PCR amplification was carried out for 28 cycles with an annealing temperature of 56°C. PCR products were visualized by staining with GelRed (Biotium, Fremont, CA, USA) and resolved on a 1.5% agarose gel. Males were identified by the presence of both the ZFX/ZFY and SRY bands, while females showed only the ZFX/ZFY band.

To assess *Borrelia* infection, we screened samples using two independent primer sets targeting different genomic regions of *Borrelia*. The first primer set amplified a 412–421 bp fragment of the ospC gene, as previously described in reference 20. The second primer set targeted the rrs-rrl (16S–23S) ribosomal intergenic spacer (21, 22). Nested PCR reactions were performed using the Type-it Microsatellite PCR Kit according to the manufacturer’s protocol. The first reaction mixture contained 1 µL of template DNA, 1 µL of PA primer (final concentration: 0.4 µM), 1 µL of P95 primer (final concentration: 0.4 µM), 12.5 µL of 2× Type-it Multiplex PCR Master Mix, and nuclease-free water up to 25 µL. The second reaction mixture contained 1 µL of PCR product from the first reaction, 1 µL of PB primer (final concentration: 0.4 µM), 1 µL of P97 primer (final concentration: 0.4 µM), 12.5 µL of 2× Type-it Multiplex PCR Master Mix, and nuclease-free water up to 25 µL. Both reactions were carried out for 30 cycles using the same annealing temperature of 52°C. PCR products were stained with GelRed and resolved on a 1.5% agarose gel. Bank voles were considered infected if a visible band of the expected size was observed with at least one of the two primer sets, though results from both sets were consistent, supporting the reliability of detection. To confirm the *Borrelia* genospecies, we Sanger sequenced the PCR amplicons obtained with the rrs-rrl primer set and compared the resulting sequences to the NCBI nucleotide database using BLAST. All sequences matched *B. afzelii*.

### RNA extraction, sequencing, and quality control

RNA was obtained from 23 infected and 21 uninfected bank vole samples and used for mRNA-seq. The samples, along with their corresponding sampling sites, body mass, sex, and *Borrelia* infection statuses, are listed in Table S1. Total RNA was extracted with the ReliaPrep RNA Tissue Miniprep System (Promega, Madison, WI, USA). RNA concentration, integrity, and purity were assessed using a 4200 TapeStation System (Agilent Technologies, Santa Clara, CA, USA) and a NanoPhotometer N120 (Implen, Munich, Germany). mRNA sequencing was performed by Novogene (Munich, Germany) using the polyA enrichment method for library preparation, followed by sequencing on the Illumina NovaSeq X Plus platform (PE150). The quality of the reads was assessed using FastQC v.0.12.1 (23), and the pre-processing of the FASTQ files, including adapter trimming and quality filtering, was performed with fastp v.0.23.4 (24).

### Expression quantification

Gene expression was estimated using Salmon v.1.10.0 (25) and the RNA FASTA file (“*_rna.fna.gz”) corresponding to annotated and accessioned RNA products from the bank vole reference genome assembly, available via the NCBI FTP site (NCBI RefSeq accession number: GCF_902806735.1) as an index. Salmon was run in quasi-mapping mode with the --validateMappings parameter enabled to improve read assignment accuracy. All the following analyses were performed in R v.4.4.1 (26). To create a table with gene counts, Salmon quantification results, and a transcript-to-gene mapping file (created from a GTF file using the rtracklayer package v.1.54.0 [27]), data were imported using the tximport package v.1.30.0 (28). Subsequently, genes with low counts (fewer than 10 reads across all samples) were filtered out. Variance stabilizing transformation was applied using the vst function from DESeq2 v. 1.30.1 (29) to generate normalized expression counts suitable for visualization and co-expression analysis.

### Differential gene expression

Differential gene expression (DGE) analysis was performed in the DESeq2 package on the filtered count data obtained from tximport. A negative binomial generalized linear model was fitted to the count data, with a design formula including infection status as a factor, along with sampling site, sex, and body mass as covariates to control for any between-population, sex, and body mass/age-related variation. To improve the interpretability and stability of fold change estimates, log_2_ fold changes (logFC) were shrunk using the apeglm method implemented in the lfcShrink() function in DESeq2. The results are presented as shrunken logFC in gene expression levels of *Borrelia*-infected bank voles compared to uninfected animals. Statistical significance was assessed using the Wald test, and *P*-values were adjusted for multiple testing using the Benjamini-Hochberg false discovery rate (FDR) procedure. Genes with an FDR < 0.05 and |logFC| > 0.58 (corresponding to approximately 1.5-fold change in gene expression) were considered significantly differentially expressed between infected and uninfected bank voles. A heatmap of gene expression for differentially expressed genes (DEGs) across all samples was generated using the pheatmap v.1.0.12 package (30).

### Weighted gene co-expression network analysis

Weighted gene co-expression network analysis (WGCNA) was performed on the normalized count data to identify potential differences in coexpressed gene clusters between infected and uninfected bank voles. Prior to the analysis, the data were adjusted for the sampling site, sex, and body mass covariates using the removeBatchEffect function from the limma v.3.58.1 package (31). WGCNA involved creating a network of genes with similar expression patterns, clustering them into modules, associating these modules with *Borrelia* infection status, and identifying driver genes in each significant module. While DGE analysis tests each gene individually and applies corrections for multiple testing, WGCNA focuses on collective expression patterns within coexpressed modules. This approach is particularly valuable for detecting more subtle but consistent within-module changes in gene expression that might not reach significance in gene-by-gene DGE analysis (32).

First, hierarchical clustering was performed using the hclust function from the fastcluster v.1.2.6 package (33) to identify potential outlier samples. Additionally, principal component analysis (PCA) was performed using the prcomp function in R to visualize sample clustering. Subsequently, WGCNA was performed using the blockwiseModules function from the WGCNA R package v.1.73 (32, 34). The adjacency matrix was constructed using Pearson’s correlation, signed network type, and a soft-threshold power determined using the scale-free topology criterion. The results of the scale-free analysis were visualized using ggplot2 v.3.5.1 (35). Modules were identified using the signed Topological Overlap Matrix and the dynamic tree cut algorithm, with a minimum module size of 50 genes. Similar modules were merged based on eigengene similarity using a cut height threshold of 0.25. The results of module clustering were visualized with the plotDendroAndColors function from the WGCNA package.

To associate the modules with *Borrelia* infection status, we correlated the module eigengenes with the infection data using Pearson’s correlation and calculated the corresponding Student’s asymptotic *P*-values using the cor and corPvalueStudent functions from the WGCNA package. The results of module-trait correlations were visualized using the pheatmap package. For subsequent analyses, we focused on modules that showed significant associations with infection status (*P* < 0.05), considering these modules as potentially biologically relevant.

To identify driver genes (hub genes) within significant modules, we performed intramodular analysis by examining module membership (MM) and gene significance (GS). MM reflects the correlation between the expression profile of an individual gene and the respective module eigengene. GS represents the correlation between individual gene expression and infection status and can have positive or negative values. We selected hub genes by applying thresholds of MM > 0.8 and |GS| > 0.2, focusing on genes with strong connections within the module and relevance to infection status.

### Functional enrichment analysis

Gene Ontology (GO) and Kyoto Encyclopedia of Genes and Genomes (KEGG) enrichment analyses were performed to explore the biological functions of DEGs and hub genes in each significant module. The set of all expressed genes identified with DESeq2 in our bank vole samples was used as the background for the analyses. GO enrichment was conducted for the biological process (BP) ontology using the Gene Annotation File for the bank vole reference genome and the topGO package v.2.46.0 (36), which applied the default weight01 algorithm and Fisher’s exact test. KEGG Orthology (KO) assignments were performed using the GhostKOALA automatic annotation server. Subsequently, the enrichKEGG function from the clusterProfiler package v.4.10.0 (37, 38) was used to perform enrichment analysis using the KO database, applying a *q*-value cutoff of 0.05.

## RESULTS

### RNA sequencing and gene expression

The sequencing of 44 samples resulted in approximately 1.52 billion read pairs. The number of read pairs per sample ranged from 29.79 million to 86.25 million, with a mean of 34.47 million read pairs per sample. After adapter trimming and quality filtering, the total number of read pairs was reduced to approximately 1.51 billion across all samples. The number of read pairs per sample after filtering ranged from 29.55 million to 85.65 million, with a mean of 34.21 million read pairs per sample. Gene expression analysis revealed 23,365 genes across all samples. After filtering out genes with low counts, 21,383 genes were retained for DGE analysis and WGCNA.

### DGE analysis

**Fig 1:**
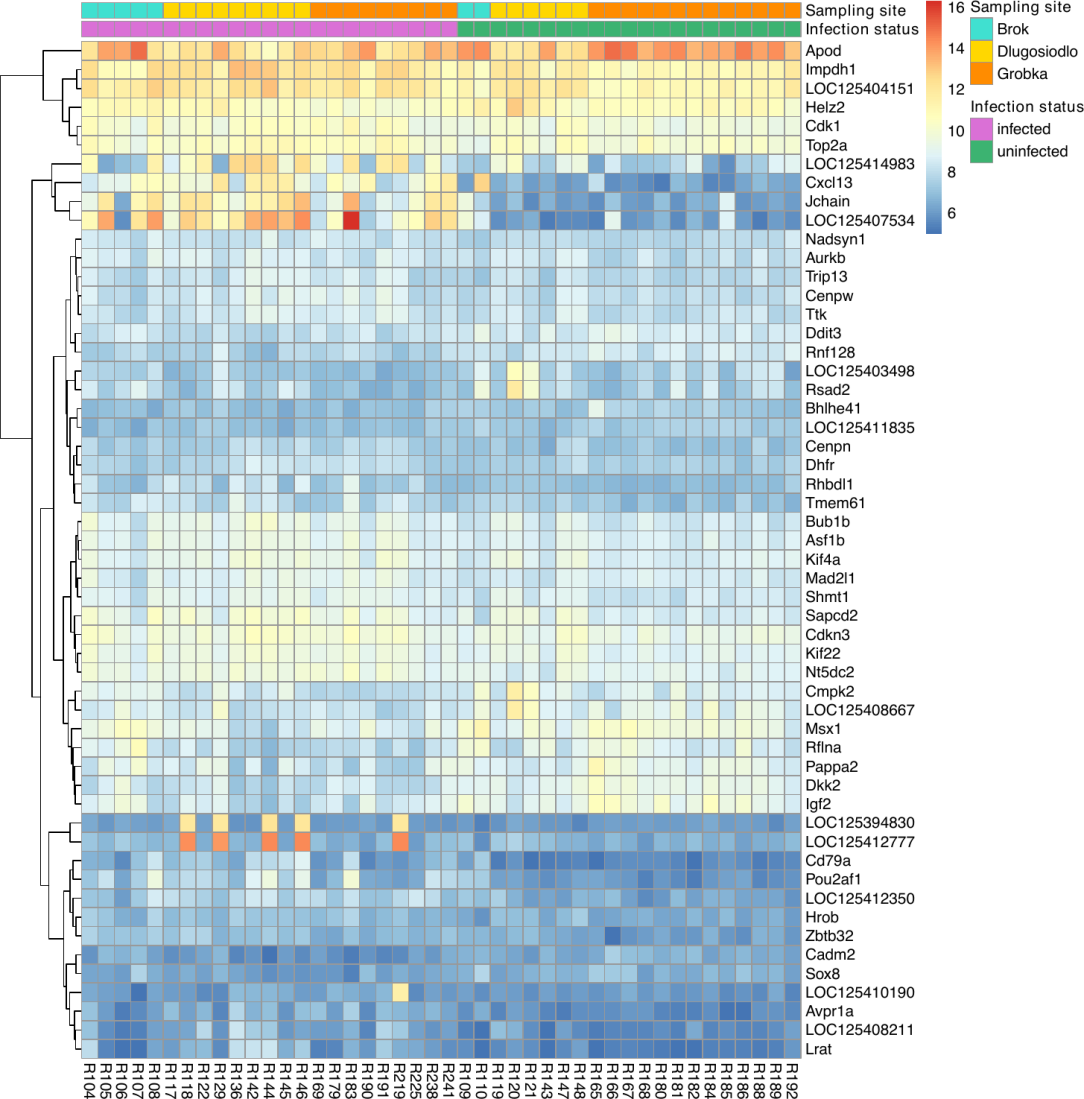
Heatmap of normalized log-transformed gene expression counts for DEGs across analyzed bank vole samples.

DGE analysis identified 54 genes with significant differential expression between infected and uninfected bank voles (Table 1). A total of 37 genes were upregulated, and 17 genes were downregulated in infected bank voles compared to uninfected ones. Normalized gene expression counts for all DEGs across analyzed samples are shown in Fig. 1. Notably, four genes—Cxcl13, Jchain, LOC125407534, and LOC125414983—showed particularly distinct expression patterns between the two bank vole groups, with all four being upregulated in infected animals (Fig. 1). The Cxcl13 gene encodes C-X-C motif chemokine ligand 13, a B lymphocyte chemoattractant that promotes B-cell migration to infection sites. The Jchain gene encodes the joining chain of multimeric IgA and IgM, a protein that regulates the polymerization of IgM and IgA antibodies. The LOC125407534 gene encodes the immunoglobulin lambda-1 light chain-like protein, a subunit of antibodies. The LOC125414983 gene encodes small proline-rich protein 2D, a cornified envelope protein of keratinocytes that also contributes to reactive oxygen species quenching and antimicrobial defense at epithelial barriers (39). These genes highlight the transcriptional differences in immune-related genes involved in B-cell recruitment and antibody function, as well as epithelial mechanisms that support defense during infection.

**TABLE 1:** DEGs between bank voles infected with *B. afzelii* and uninfected ones^*a*^

Gene	Gene description	baseMean	logFC	lfcSE	*P* value	*P*adj
LOC125407534	Immunoglobulin lambda-1 light chain-like	4,810.85	5.69	1.15	8.82E−14	1.26E−09
Jchain	Joining chain of multimeric IgA and IgM	1,546.42	3.69	0.84	1.84E−10	1.32E−06
Lrat	Lecithin retinol acyltransferase	36.53	3.44	0.75	3.20E−07	0.002
LOC125412777	40S ribosomal protein S2	2,432.67	3.68	1.27	2.82E−06	0.010
LOC125412350	Cyclin-dependent kinase inhibitor 2A-like	102.86	1.48	0.37	4.37E−06	0.013
Bhlhe41	Basic helix-loop-helix family member e41	104.42	−1.00	0.27	6.52E−06	0.016
Kif22	Kinesin family member 22	579.09	0.65	0.18	9.36E−06	0.019
LOC125394830	40S ribosomal protein S2-like	485.49	3.17	1.10	1.22E−05	0.019
Cenpn	Centromere protein N	121.59	0.84	0.24	1.39E−05	0.020
LOC125410190	Keratin, type II cytoskeletal 5-like	88.78	2.71	0.76	1.96E−05	0.020
LOC125411835	Zinc finger protein 54-like	96.49	−0.73	0.21	1.55E−05	0.020
Cmpk2	Cytidine/uridine monophosphate kinase 2	435.97	−1.10	0.32	3.74E−05	0.023
Dkk2	Dickkopf WNT signaling pathway inhibitor 2	403.65	−0.93	0.30	3.45E−05	0.023
LOC125403498	Interferon-induced protein with tetratricopeptide repeats 2-like	186.46	−1.44	0.45	3.43E−05	0.023
Nadsyn1	NAD synthetase 1	221.92	0.60	0.19	3.99E−05	0.023
Nt5dc2	5′-nucleotidase domain containing 2	578.79	0.66	0.19	3.38E−05	0.023
Pou2af1	POU class two homeobox associating factor 1	129.22	1.83	0.75	3.76E−05	0.023
Rnf128	Ring finger protein 128	220.65	−0.81	0.25	3.37E−05	0.023
Dhfr	Dihydrofolate reductase	158.75	0.65	0.20	4.21E−05	0.023
Cdkn3	Cyclin-dependent kinase inhibitor 3	777.45	0.64	0.20	4.79E−05	0.025
Shmt1	Serine hydroxymethyltransferase 1	383.14	0.61	0.20	5.67E−05	0.029
Hrob	Homologous recombination factor with OB-fold	62.92	0.93	0.32	7.04E−05	0.034
Sapcd2	Suppressor APC domain containing 2	599.74	0.83	0.27	7.19E−05	0.034
Asf1b	Anti-silencing function 1B histone chaperone	448.20	0.59	0.19	7.92E−05	0.035
LOC125408667	Guanylate-binding protein 6-like	468.77	−1.14	0.37	9.06E−05	0.037
Tmem61	Transmembrane protein 61	164.79	0.98	0.33	8.82E−05	0.037
LOC125408211	Transmembrane protease serine 11G	35.18	2.05	0.73	1.04E−04	0.040
Mad2l1	Mitotic arrest deficient 2 like 1	388.28	0.61	0.21	1.05E−04	0.040
LOC125414983	Small proline-rich protein 2D	1,280.92	2.15	0.69	1.11E−04	0.041
Msx1	msh homeobox 1	689.89	−0.86	0.30	1.27E−04	0.041
Rsad2	Radical S-adenosyl methionine domain containing 2	265.09	−1.54	0.53	1.30E−04	0.041
Apod	Apolipoprotein D	11,417.49	−0.95	0.36	1.44E−04	0.042
Bub1b	BUB1 mitotic checkpoint serine/threonine kinase B	433.60	0.67	0.24	1.41E−04	0.042
Kif4a	Kinesin family member 4A	500.28	0.61	0.21	1.45E−04	0.042
Avpr1a	Arginine vasopressin receptor 1A	29.44	1.73	0.56	1.78E−04	0.045
Ddit3	DNA damage inducible transcript 3	282.82	−0.59	0.20	2.32E−04	0.045
Helz2	Helicase with zinc finger 2	1,798.68	−0.63	0.22	2.29E−04	0.045
Impdh1	Inosine monophosphate dehydrogenase 1	3,675.74	0.79	0.29	2.09E−04	0.045
LOC125404151	Ultra-long-chain fatty acid omega-hydroxylase	3,476.62	0.69	0.25	1.91E−04	0.045
Rflna	Refilin A	391.20	−0.96	0.40	2.21E−04	0.045
Rhbdl1	Rhomboid like 1	126.60	0.96	0.35	2.23E−04	0.045
Sox8	SRY-box transcription factor 8	44.91	−1.08	0.41	2.32E−04	0.045
Top2a	DNA topoisomerase II alpha	1,275.08	0.58	0.22	2.01E−04	0.045
Ttk	TTK protein kinase	273.54	0.62	0.23	1.99E−04	0.045
Cadm2	Cell adhesion molecule 2	33.05	−1.23	0.48	3.03E−04	0.048
Cd79a	CD79a molecule	56.60	1.49	0.77	2.93E−04	0.048
Cdk1	Cyclin-dependent kinase 1	1,043.07	0.63	0.24	2.85E−04	0.048
Cenpw	Centromere protein W	273.32	0.68	0.25	2.73E−04	0.048
Cxcl13	C-X-C motif chemokine ligand 13	921.69	2.12	0.88	2.98E−04	0.048
Igf2	Insulin-like growth factor 2	520.61	−0.88	0.35	2.75E−04	0.048
Aurkb	Aurora kinase B	288.12	0.59	0.23	3.08E−04	0.048
Zbtb32	Zinc finger and BTB domain containing 32	57.45	0.87	0.36	3.22E−04	0.049
Pappa2	Pappalysin 2	446.56	−0.95	0.47	3.27E−04	0.049
Trip13	Thyroid hormone receptor interactor 13	235.18	0.67	0.27	3.31E−04	0.049

^*a*^ logFC represents log_2_ fold change in gene expression between infected and uninfected voles, where a positive logFC value indicates that a gene is upregulated in infected voles compared to uninfected ones, while a negative logFC value indicates that a gene is downregulated in infected voles compared to uninfected. baseMean: the average normalized expression level of each gene across all samples. lfcSE: the standard error of the log_2_ fold change estimate. *P *value: raw *P*-value from the test. *P*adj: Benjamini-Hochberg adjusted *P*-value (FDR).

Enrichment analysis of DEGs identified multiple significant GO BPs (Table S2) and two KEGG pathways: cell cycle (map04110, *q* = 1.15E−04) and biosynthesis of cofactors (map01240, *q* = 0.042). Among the enriched GO terms, several were related to the immune response. The defense response to virus (GO:0051607, *P* = 0.009) included downregulated genes, such as LOC125403498, which encodes an interferon-induced protein with tetratricopeptide repeats 2-like, and Rsad2, which encodes an interferon-inducible antiviral protein. The defense response to protozoan (GO:0042832, *P* = 0.041) included downregulated LOC125408667, which encodes guanylate-binding protein 6-like, an interferon-inducible GTPase that plays a role in the innate immune response. The cellular response to biotic stimulus (GO:0071216, *P* = 0.012) included upregulated Cxcl13 and downregulated Ddit3, which encodes the DNA damage inducible transcript 3, a multifunctional transcription factor involved in response to cell stress and apoptosis. Last, the peptide antigen assembly with major histocompatibility complex (MHC) class II protein complex (GO:0002503, *P* = 0.038) included upregulated LOC125407534, which encodes an immunoglobulin lambda-1 light chain like.

The enrichment analysis also identified several GO terms associated with metabolic processes, such as the glycine metabolic process (GO:0006544, *P* = 5.4E−04), the water-soluble vitamin metabolic process (GO:0006767, *P* = 0.001), and the NAD biosynthetic process (GO:0009435, *P* = 0.038). Furthermore, GO enrichment highlighted terms associated with cell cycle regulation, chromosome segregation, gene expression, development, and morphogenesis.

### Weighted gene co-expression network analysis

**Fig 2:**
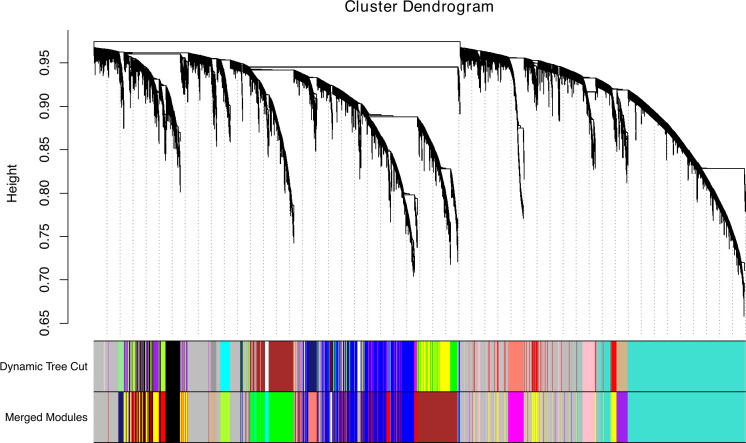
Hierarchical clustering dendrogram of genes, constructed to identify co-expression modules. Different colors represent different modules, with gray indicating unassigned genes. The dynamic tree cut represents the initial clustering, while the final module assignment, based on eigengene similarity, is shown as merged modules.

**Fig 3:**
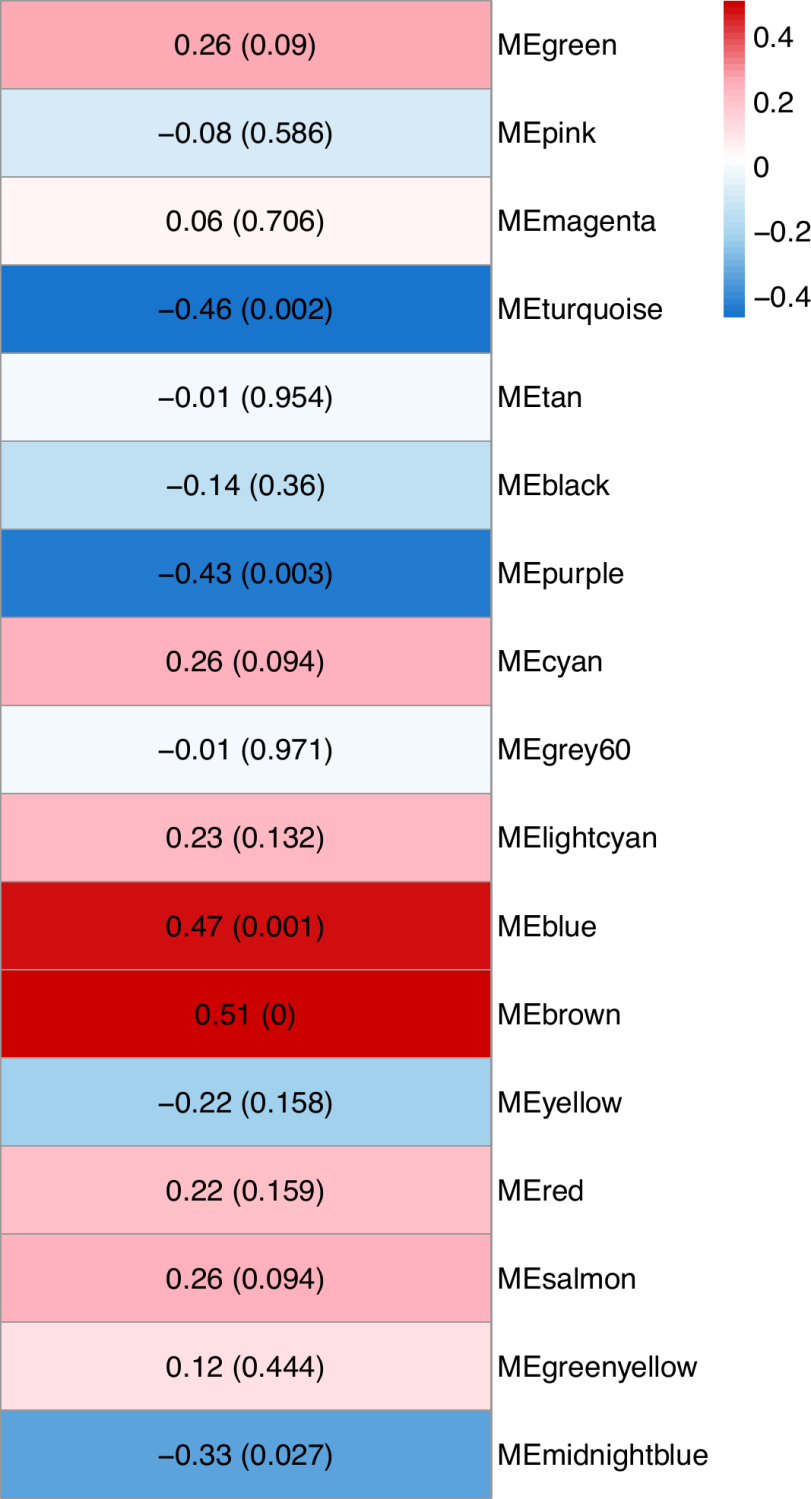
Module-trait correlation heatmap visualizing the correlation between WGCNA module eigengenes and *B. afzelii* infection status. Each row represents a module, and each cell contains the correlation coefficient with the corresponding *P*-value in parentheses. Red indicates a positive correlation between the module and infection, while blue indicates a negative correlation.

Based on the hierarchical clustering dendrogram of samples (Fig. S1) and the PCA plot (Fig. S2), no clear outliers were detected. For WGCNA, a soft threshold power of 6 was selected based on the scale-free topology model fit (Fig. S3). WGCNA identified 17 gene modules, represented by different colors, excluding the gray module, which represents unassigned genes. The cluster dendrogram of the detected modules is shown in Fig. 2. Of the modules discovered, five were significantly correlated with infection status (*P* < 0.05; Fig. 3), including the blue, brown, turquoise, purple, and midnight blue modules. The blue and brown modules contained 2,509 and 1,793 genes, respectively, and were positively correlated with infection, meaning that the genes in these modules had higher expression levels in response to infection. The turquoise, purple, and midnight blue modules contained 4,313, 486, and 167 genes, respectively, and were negatively correlated with infection. After selecting hub genes based on their MM and GS, the gene counts were as follows: 325 in the blue module, 306 in the brown, 629 in the turquoise, 65 in the purple, and 22 in the midnight blue.

Functional enrichment analysis revealed GO terms and KEGG pathways enriched in the hub genes present in the significant modules (Tables S3 to S10). Two modules that were positively correlated with infection status, the blue and brown modules, showed significant enrichment in BPs related to defense response to bacteria, metabolism, energy production, regulation of gene expression, protein synthesis and folding, neuronal functions, transport and signaling, as well as cell cycle, apoptosis, and actin filament organization (Tables S3 and S4). GO terms associated with defense responses and apoptosis included genes involved in programmed cell death, such as the Gsdma gene, which encodes gasdermin A, and LOC125406092, which encodes NLR family apoptosis inhibitory protein. Terms related to metabolism covered various functions, encompassing carbohydrate, lipid, amino acid, and nucleotide metabolism, with the glycolytic process (GO:0006096, *P* = 5.4E−10) being the most significant term. Additionally, KEGG enrichment identified one pathway in the blue module: the cornified envelope formation (map04382, *q* = 2.46E−11) and several pathways within the brown module that were associated with neurodegenerative diseases, such as amyotrophic lateral sclerosis, prion disease, Parkinson’s, Huntington’s, and Alzheimer’s disease. Other enriched pathways in the brown module were related to metabolism and energy production, infectious diseases (*E. coli* infection, *Salmonella* infection, and legionellosis), and cellular processes and signaling, including the HIF-1 signaling pathway, p53 signaling pathway, and necroptosis (Table S5).

Three modules that were negatively correlated with infection status, the turquoise, purple, and midnight blue modules, were enriched in GO terms related to extracellular matrix organization, cell migration, and adhesion. Furthermore, enriched terms were associated with gene expression, protein processing, tissue development, angiogenesis, nervous system development, myelination, and signaling (Tables S6 to S8). Notably, the midnight blue module included several immune-related GO terms such as defense response to virus, innate immune response, and regulation of cytokine production (Table S8). Moreover, several significant KEGG pathways were identified in the turquoise module, including those related to cell adhesion, intracellular signaling, and hormone regulation, as well as pathways associated with various cancers, cardiovascular diseases, and infectious diseases, all of which can be linked to changes in cell signaling and adhesion (Table S9). Additionally, the midnight blue module was enriched in pathways related to innate antiviral immune responses (Table S10).

## DISCUSSION

In the present study, we explored changes in gene expression in wild bank vole populations in response to *B. afzelii* infection, with a focus on the skin of the ear as the infection site. While the data may be noisy due to the complexities of studying wild animals, including environmental factors, different stages of *Borrelia* infection, and potential coinfections with other pathogens, it provides valuable insights into host-pathogen interactions within a natural ecological context.

We conducted DGE analysis and WGCNA, followed by enrichment analysis, and uncovered a broad set of genes and pathways involved in the bank vole response to *B. afzelii* infection. Our findings highlighted diverse responses of the bank vole transcriptome, as indicated by 54 DEGs and five gene modules correlated with infection. The results of our analysis revealed several immune-related genes, as well as numerous genes involved in metabolic processes, cellular structure, gene expression, and cell cycle regulation.

Notably, most immune-related genes were associated with B-cell activity and antibody production. For example, we identified upregulated antibody-related genes: Jchain and immunoglobulin lambda-1 light chain like, as well as Cxcl13, which encodes a B-cell chemoattractant, the presence of which in cerebrospinal fluid is known to be a marker of neuroborreliosis (40, 41). Moreover, among upregulated DEGs, we found the Cd79a gene, which encodes a component of the B-cell receptor complex. These results highlight the role of adaptive immune response to infection with *Borrelia*, consistent with the findings of high seroprevalence and association of strain-specific infections with MHC genotype (20, 42).

Studies in humans with Lyme disease report a strong pro-inflammatory response to *Borrelia* infection, including upregulation of genes encoding interferons, pro-inflammatory cytokines (TNF, IL1B, and IL6), toll-like receptor 2 (TLR2), as well as C-C and C-X-C motif chemokines (6, 43, 44). In contrast, we did not observe such a pronounced pro-inflammatory immune response in bank voles. Notably, genes induced by type I interferons, such as LOC125403498 (encoding an Ifit2-like protein) and Rsad2, were downregulated. Additionally, Ifit3 was in the midnight blue WGCNA module, negatively correlated with infection. This is particularly interesting, as type I interferons and interferon-stimulated genes, such as Ifit2, Ifit3, or Rsad2, have previously been shown to be upregulated in erythema migrans skin lesions from untreated patients with Lyme disease (43).

Notably, in our analysis, TNF superfamily member 12 (Tnfsf12), a pro-inflammatory and pro-apoptotic cytokine (45, 46), was found in the turquoise module, which was negatively correlated with infection. In addition, two receptors for pro-inflammatory cytokines (Il11ra and Il17rd) were also present in the turquoise module, further suggesting a suppression of inflammation in bank voles. In the brown module, which was positively correlated with infection, we also found the interleukin-1 receptor antagonist (Il1rn), which inhibits the activities of pro-inflammatory cytokines IL1A and IL1B. These results are consistent with the idea that responses in reservoir hosts, such as bank voles, differ from responses in non-competent, accidental hosts, such as humans, whereby reservoir hosts regulate their immune responses to prevent excessive inflammation and tissue damage. This modulation may explain why Zhong et al. (47) did not observe pathological changes in infected bank vole joints similar to those seen in human Lyme disease, despite *Borrelia* dissemination to joints occurring in a larger proportion of infected bank voles.

Another potential mechanism by which bank voles may modulate *Borrelia* infection, limit its dissemination, and prevent the establishment of chronic infection is through changes in the expression of genes related to the extracellular matrix. *Borrelia* is known to bind to laminin (48) and colonize and degrade collagen fibers, facilitating its spread and invasion into connective tissues of various organs, such as joints, heart, and nervous system (49). In line with this, we found that the expression of bank vole genes encoding collagens (Col8a1, Col8a2, Col16a1, and Col28a1) and laminins (Lama2, Lama4, Lamb2, and Lamc1) was negatively correlated with *B. afzelii* infection. Interestingly, a study on human dermal fibroblasts found that genes encoding collagen (Col8a1) and laminin (Lama1) were upregulated by *Borrelia* (50). An additional example is matrix metalloproteinases (MMPs), enzymes involved in the degradation of the extracellular matrix, influencing processes such as tissue remodeling. We found matrix metallopeptidases (Mmp2, Mmp14, and Mmp17) in the turquoise module, which was negatively correlated with infection, while studies in humans reported elevated levels of different MMPs in response to *Borrelia* (50–52). These differences highlight the distinct responses of reservoir hosts and accidental end-point hosts, which may have consequences for different courses of infection: while reservoirs do not exhibit clinical manifestations of *Borrelia* infection (53, 54), accidental hosts such as humans often develop chronic multi-organ disease with symptoms including fatigue, fever, headaches, joint pain and swelling, heart palpitations, and neurological disorders (7).

We also identified several metabolic and mitochondrial pathways upregulated in both the DGE analysis and WGCNA, particularly in the brown module, which was positively correlated with infection. Enriched GO terms included glycolysis, water-soluble vitamin metabolism, oxidative phosphorylation, ATP synthesis, protein folding, and pathways related to protein and electron transport. These enrichments indicate that *B. afzelii* infection is associated with shifts in cellular energy metabolism and mitochondrial activity, potentially reflecting increased energetic demands during infection or the metabolic dependence of *Borrelia* on host-derived nutrients (6, 55).

Our results are broadly consistent with the findings of previous studies on the transcriptional responses of reservoir hosts to *Borrelia*, confirming key patterns observed in earlier research (13–16). A previous study that compared the spleen transcriptome responses of *B. afzelii* infected vs uninfected wild bank voles identified eight DEGs and 28 Hallmark gene sets uncovered by gene set enrichment analysis, including upregulated processes such as oxidative phosphorylation and heme metabolism, as well as downregulated processes, such as inflammatory response, TGF beta signaling, and Notch signaling (13), which were also identified by our enrichment analysis. Additionally, among the downregulated Hallmark gene sets identified by Zhong et al. (13), there was the epithelial-mesenchymal transition set, which is involved in wound healing and fibrosis. Similarly, our analysis revealed downregulation of GO terms and KEGG pathways related to extracellular matrix organization, involving genes such as laminins, collagens, and metalloproteinases, suggesting shared changes in skin tissue as a potential defensive mechanism to restrict *Borrelia* dissemination. However, by focusing on the skin as the infection site, we identified more DEGs compared to the analysis of the spleen transcriptome, which is consistent with the findings of a previous study of white-footed mice (*P. leucopus*) experimentally infected with *B. burgdorferi* s.s., which found that during persistent infection, more DEGs were present in the skin than in the blood (15). The study by Long et al. (15), Bourgeois et al. (16), and another experimental study by Gaber et al. (14), which compared spleen transcriptome responses to *Borrelia* infection in *P. leucopus* and mice (*M. musculus*), showed similar transcriptional patterns in response to infection as observed in our results. The study by Long et al. revealed that upregulated DEGs in the skin included immunoglobulin light chain genes and several keratin genes, while downregulated genes were related to the cytoskeleton or extracellular matrix, such as laminin, similar to our findings. They also found no genes associated with inflammation, which aligns with our results. The study by Bourgeois et al. (16) reported strong upregulation of Cxcl13 and Jchain in *P. leucopus* skin at 8 weeks post-infection, and we similarly observed differential expression of these genes between infected and uninfected voles. The study by Gaber et al. identified pathways such as hydroxycarboxylic acid-binding receptors, auto-degradation of Cdh1 by Cdh1:APC/C, hepatitis C, protein processing in the endoplasmic reticulum, adipocytokine signaling pathway, and peroxisome proliferator-activated receptor signaling pathway. Our results are similar as we identified several metabolic pathways, cell cycle regulation processes, and immune responses to infection. These results support a shared biological response to *Borrelia* infection, reflecting both metabolic and immunoregulatory mechanisms.

It is important to note that some changes in bank vole gene expression may not be caused by *Borrelia* itself, but rather by tick saliva, which is necessary to establish *Borrelia* infection (17, 56), or by other pathogens co-transmitted by ticks, such as *Bartonella* or *Rickettsia* (57). However, our findings are consistent with previous experimental studies discussed earlier, including the result of an experiment in which *P. leucopus* were infected via inoculation (15), bypassing the influence of tick saliva and avoiding co-infection. This suggests that *Borrelia* itself was a major factor altering host gene expression that we observed. Furthermore, despite the non-negligible co-infection frequency in European rodents reported by Galfsky et al. (57), the degree of overlap between *Borrelia* infection and infections by other tick-borne species is low. For example, the three most common tick-transmitted pathogens found in the rodent sample (*Bartonella* spp., *Candidatus Neoehrlichia mikurensis*, and *Rickettsia* spp.; prevalence 78.2%, 58.2%, and 29.1% of rodents, respectively) were simultaneously detected in only 19 of 80 (24%) rodents infected with *Borrelia* (see Table 4 in reference 57). Therefore, it seems unlikely that the immune response we observed could be explained by the confounding effects of these co-infections.

We also acknowledge that we might have failed to detect infection in our tissue samples in some cases, particularly when the infection level was low. However, low-level infection elicits a weak immune response, so such false negative cases should not have much of an effect on our DGE results. Indeed, the fact that our analysis revealed highly significant overexpression of key genes known to be associated with *Borrelia* infection gives us confidence that our assignment to infected vs uninfected groups was largely accurate.

Studies on bank voles reported no severe effects on their condition and survival (11). An effective immune response, including the adaptive immune responses at the site of infection evident in our data, may contribute to controlling infection. Indeed, earlier work on voles and *Peromyscus* indicates that anti-*Borrelia* antibodies are present in a larger proportion of rodents than those actively infected at the time of sampling (20, 58), suggesting that infection can be effectively cleared. However, effective clearance may involve costs, as experimental infection with *Borrelia* altered the reproductive success of bank voles, particularly in males in a low-density environment, where increased exploratory behavior is required (12). In our study, infection was associated with alterations in metabolic pathways, suggesting that host responses may involve shifts in cellular energy allocation that reflect the energetic demands of the immune response.

To conclude, our results revealed the involvement of numerous genes related to immune responses, metabolism, cellular structure, gene expression, and cell cycle regulation, which aligns with previous studies on rodents (13–16). We observed upregulation of pathways related to B-cell activity and antibody production, along with shifts in cellular energy metabolism and changes in pathways related to extracellular matrix composition. These findings provide new insights into host-pathogen interactions in reservoir hosts and offer potential directions for further investigation into the molecular mechanisms underlying *Borrelia* infection.

### Highlights

- RNA-seq was used to investigate the transcriptomic response of wild bank voles to infection with *B. afzelii* at the site of entry, the ear skin.
- A total of 37 upregulated and 17 downregulated DEGs were identified in infected bank voles compared to uninfected ones.
- Five WGCNA gene modules were positively or negatively correlated with infection status.
- The infected bank voles showed differences in the expression of genes related to immune response, metabolism, gene expression, cell cycle regulation, and extracellular matrix organization.

## Data Availability

The data have been deposited in the Open Science Framework (OSF) repository and are publicly available at https://osf.io/4deyj. The Illumina sequencing data are available in the NCBI Sequence Read Archive under accession number PRJNA1298558.

## SUPPLEMENTAL MATERIAL

Supplemental figures spectrum.02574-25-s0001.pdf Figures S1 to S3.

Supplemental material spectrum.02574-25-s0002.csv Full DESeq2 output.

Supplemental tables spectrum.02574-25-s0003.pdf Tables S1 to S10.

ASM does not own the copyrights to Supplemental Material that may be linked to, or accessed through, an article. The authors have granted ASM a non-exclusive, world-wide license to publish the Supplemental Material files. Please contact the corresponding author directly for reuse.
