# Supplementary material for: Transcriptomic response to Borrelia afzelii infection in the skin of wild bank voles
Source: Microbiol Spectr. 2026 Jan 27;14(3):e02574-25. doi: 10.1128/spectrum.02574-25 (PMC12955458; doi:10.1128/spectrum.02574-25)
Supplement: Supplemental figures — Figures S1 to S3. [file spectrum.02574-25-s0001.pdf]

Supplementary Figures

Transcriptomic response to *Borrelia afzelii* infection in the skin of wild bank voles

Joanna Różańska-Wróbel[1], Mateusz Konczal[1], Rocco F. Notarnicola[1], Jacek Radwan[1]

[1] Evolutionary Biology Group, Institute of Environmental Biology, Faculty of Biology, Adam Mickiewicz University, Poznań, Poland

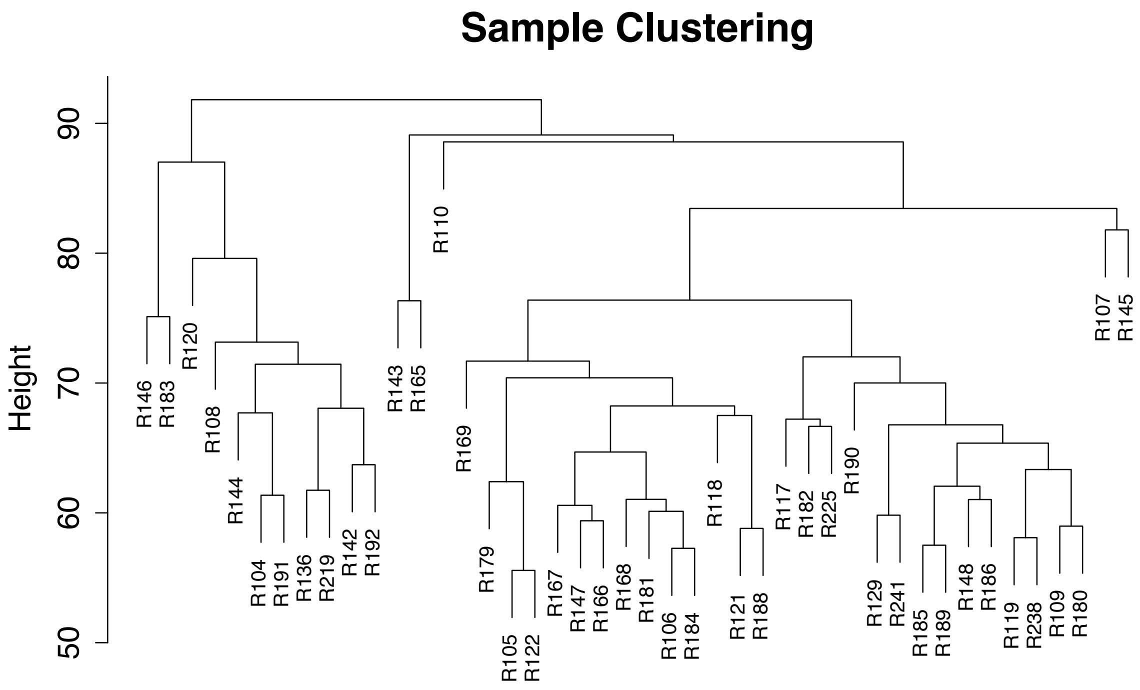

Figure S1. Hierarchical clustering dendrogram of samples based on normalized gene expression profiles, performed to identify potential outliers. No outlier samples were detected.

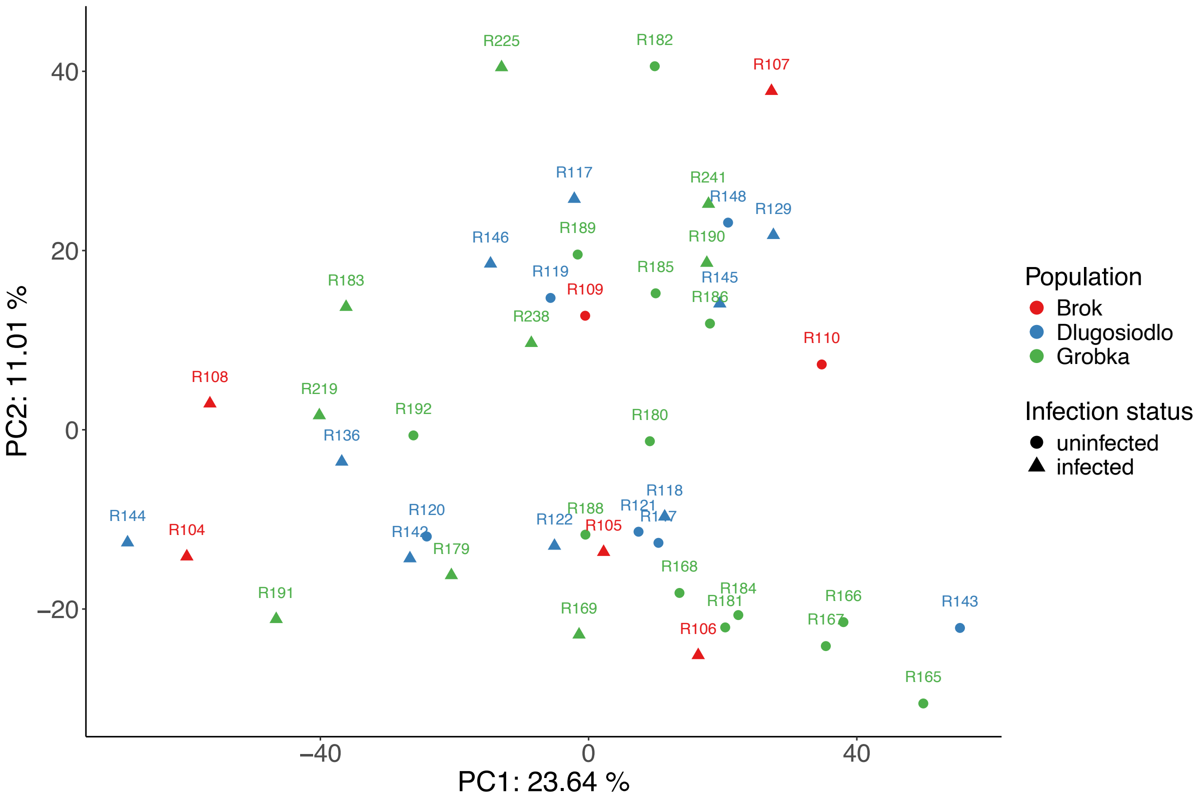

Figure S2. Principal component analysis (PCA) of normalized gene expression data across samples. Each point represents a bank vole sample, colored by population (Brok, Długosiodło, or Grobka) and shaped by *B. afzelii* infection status (infected or uninfected).

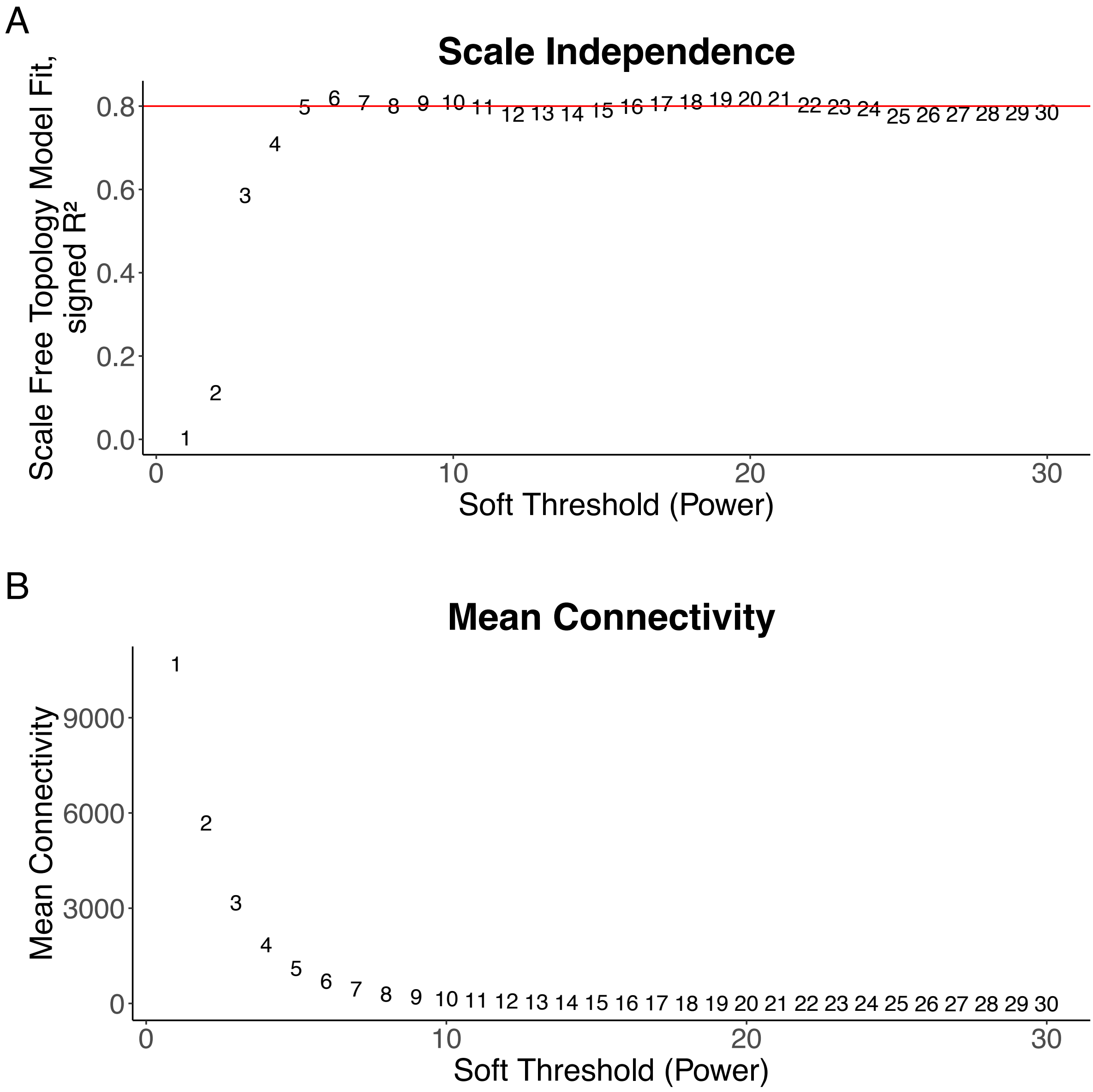

Figure S3. Scale-free topology analysis under different soft thresholding powers (1 – 30) performed to determine the optimal power for WGCNA network construction: A) scale-free topology model fit; B) mean connectivity. A power of 6 was chosen, resulting in an R^2 of 0.82, which ensures a reasonable approximation to scale-free topology, with a mean connectivity of 707.
