# Supplementary material for: Transcriptomic response to Borrelia afzelii infection in the skin of wild bank voles
Source: Microbiol Spectr. 2026 Jan 27;14(3):e02574-25. doi: 10.1128/spectrum.02574-25 (PMC12955458; doi:10.1128/spectrum.02574-25)
Supplement: Supplemental tables — Tables S1 to S10. [file spectrum.02574-25-s0003.pdf]

Supplementary Tables

Transcriptomic response to *Borrelia afzelii* infection in the skin of wild bank voles

Joanna Różańska-Wróbel[1], Mateusz Konczal[1], Rocco F. Notarnicola[1], Jacek Radwan[1]

[1] Evolutionary Biology Group, Institute of Environmental Biology, Faculty of Biology, Adam Mickiewicz University, Poznań, Poland

Table S1. Bank vole samples used for RNA sequencing.

Bank vole ID	Sampling site	Body mass [g]	sex	*B. afzelii* infection status
R104	Brok	21	female	infected
R105	Brok	25	female	infected
R106	Brok	12	male	infected
R107	Brok	22	male	infected
R108	Brok	21	male	infected
R109	Brok	21	female	uninfected
R110	Brok	23	female	uninfected
R117	Długosiodło	24	female	infected
R118	Długosiodło	26	female	infected
R119	Długosiodło	17	female	uninfected
R120	Długosiodło	22	female	uninfected
R121	Długosiodło	16	female	uninfected
R122	Długosiodło	25	male	infected
R129	Długosiodło	20	male	infected
R136	Długosiodło	25	female	infected
R142	Długosiodło	24	female	infected
R143	Długosiodło	15	female	uninfected
R144	Długosiodło	23	male	infected
R145	Długosiodło	24	female	infected
R146	Długosiodło	23	male	infected
R147	Długosiodło	12	female	uninfected
R148	Długosiodło	11	female	uninfected
R165	Grobka	17	male	uninfected
R166	Grobka	26	female	uninfected
R167	Grobka	19	male	uninfected
R168	Grobka	18	female	uninfected
R169	Grobka	16	male	infected
R179	Grobka	20.5	male	infected
R180	Grobka	16	male	uninfected
R181	Grobka	16	male	uninfected
R182	Grobka	16	male	uninfected
R183	Grobka	19	male	infected

R184	Grobka	17	female	uninfected
R185	Grobka	18	female	uninfected
R186	Grobka	15.5	female	uninfected
R188	Grobka	18	female	uninfected
R189	Grobka	17	male	uninfected
R190	Grobka	18	male	infected
R191	Grobka	18.5	male	infected
R192	Grobka	15.5	male	uninfected
R219	Grobka	17.5	male	infected
R225	Grobka	20.5	female	infected
R238	Grobka	25.5	male	infected
R241	Grobka	21	male	infected

Table S2. Summary of GO biological process enrichment analysis of differentially expressed genes.

GO ID	Term	Annotated	Significant	Expected	weightedFisher
GO:0007094	mitotic spindle assembly checkpoint signaling	14	3	0.04	1.10E-05
GO:0051445	regulation of meiotic cell cycle	10	2	0.03	4.40E-04
GO:0006544	glycine metabolic process	11	2	0.04	5.40E-04
GO:0007052	mitotic spindle organization	52	3	0.17	6.00E-04
GO:0046653	tetrahydrofolate metabolic process	13	2	0.04	7.60E-04
GO:0009070	serine family amino acid biosynthetic process	13	2	0.04	7.60E-04
GO:0007059	chromosome segregation	140	6	0.45	0.0011
GO:0006767	water-soluble vitamin metabolic process	16	2	0.05	0.0012
GO:0000075	cell cycle checkpoint signaling	60	5	0.19	0.0029
GO:0043648	dicarboxylic acid metabolic process	34	2	0.11	0.0052
GO:0000819	sister chromatid segregation	80	4	0.26	0.0060
GO:0051607	defense response to virus	44	2	0.14	0.0086
GO:0051784	negative regulation of nuclear division	17	4	0.05	0.0089
GO:0051307	meiotic chromosome separation	15	2	0.05	0.0093
GO:0043603	cellular amide metabolic process	948	5	3.02	0.0094
GO:0071216	cellular response to biotic stimulus	48	2	0.15	0.0125
GO:0006334	nucleosome assembly	56	2	0.18	0.0137
GO:0072522	purine-containing compound biosynthetic process	106	2	0.34	0.0249
GO:0006984	ER-nucleus signaling pathway	10	1	0.03	0.0315
GO:0007095	mitotic G2 DNA damage checkpoint signaling	10	1	0.03	0.0315
GO:0009263	deoxyribonucleotide biosynthetic process	10	1	0.03	0.0315
GO:0070059	intrinsic apoptotic signaling pathway in response to endoplasmic reticulum stress	10	1	0.03	0.0315
GO:0042246	tissue regeneration	10	1	0.03	0.0315
GO:0060348	bone development	10	1	0.03	0.0315

GO:0042559	pteridine-containing compound biosynthetic process	10	1	0.03	0.0315
GO:0051382	kinetochore assembly	10	1	0.03	0.0315
GO:0003044	regulation of systemic arterial blood pressure mediated by a chemical signal	11	1	0.04	0.0345
GO:0006183	GTP biosynthetic process	11	1	0.04	0.0345
GO:0006144	purine nucleobase metabolic process	11	1	0.04	0.0345
GO:0050886	endocrine process	11	1	0.04	0.0345
GO:0009071	serine family amino acid catabolic process	11	1	0.04	0.0345
GO:0031055	chromatin remodeling at centromere	11	1	0.04	0.0345
GO:0009394	2'-deoxyribonucleotide metabolic process	11	1	0.04	0.0345
GO:0000712	resolution of meiotic recombination intermediates	12	1	0.04	0.0376
GO:0001523	retinoid metabolic process	12	1	0.04	0.0376
GO:0002503	peptide antigen assembly with MHC class II protein complex	12	1	0.04	0.0376
GO:0009435	NAD biosynthetic process	12	1	0.04	0.0376
GO:0006730	one-carbon metabolic process	12	1	0.04	0.0376
GO:0034508	centromere complex assembly	23	2	0.07	0.0398
GO:0009148	pyrimidine nucleoside triphosphate biosynthetic process	13	1	0.04	0.0407
GO:0030282	bone mineralization	13	1	0.04	0.0407
GO:0042832	defense response to protozoan	13	1	0.04	0.0407
GO:0019886	antigen processing and presentation of exogenous peptide antigen via MHC class II	13	1	0.04	0.0407
GO:2001235	positive regulation of apoptotic signaling pathway	14	1	0.04	0.0438
GO:0030261	chromosome condensation	15	1	0.05	0.0468
GO:0046112	nucleobase biosynthetic process	15	1	0.05	0.0468
GO:0051262	protein tetramerization	16	1	0.05	0.0499

GO:0048705	skeletal system morphogenesis	16	1	0.05	0.0499
GO:0032922	circadian regulation of gene expression	16	1	0.05	0.0499
GO:0034502	protein localization to chromosome	16	1	0.05	0.0499

Table S3. Summary of GO biological process enrichment analysis of the blue gene module identified by WGCNA.

GO ID	Term	Annotated	Significant	Expected	weightedFisher
GO:0046475	glycerophospholipid catabolic process	15	5	0.26	4.20E-06
GO:0012501	programmed cell death	324	10	5.71	1.10E-05
GO:0006508	proteolysis	996	34	17.54	2.80E-05
GO:0046519	sphingoid metabolic process	11	3	0.19	8.00E-04
GO:0016192	vesicle-mediated transport	632	19	11.13	9.70E-04
GO:0042742	defense response to bacterium	71	8	1.25	0.002
GO:0071577	zinc ion transmembrane transport	17	3	0.3	0.003
GO:0019731	antibacterial humoral response	19	3	0.33	0.004
GO:0050773	regulation of dendrite development	18	4	0.32	0.004
GO:0010951	negative regulation of endopeptidase activity	63	5	1.11	0.005
GO:0043281	regulation of cysteine-type endopeptidase activity involved in apoptotic process	37	3	0.65	0.008
GO:0009395	phospholipid catabolic process	24	7	0.42	0.010
GO:0051650	establishment of vesicle localization	50	5	0.88	0.010
GO:0010769	regulation of cell morphogenesis involved in differentiation	10	2	0.18	0.013
GO:0018149	peptide cross-linking	10	2	0.18	0.013
GO:0006506	GPI anchor biosynthetic process	28	3	0.49	0.013
GO:0030050	vesicle transport along actin filament	11	2	0.19	0.015
GO:0002920	regulation of humoral immune response	11	2	0.19	0.015
GO:0034332	adherens junction organization	30	3	0.53	0.015
GO:0009063	cellular amino acid catabolic process	56	4	0.99	0.017
GO:0046513	ceramide biosynthetic process	31	3	0.55	0.017
GO:0030517	negative regulation of axon extension	21	2	0.37	0.018
GO:0071108	protein K48-linked deubiquitination	12	2	0.21	0.018

GO:0048814	regulation of dendrite morphogenesis	12	2	0.21	0.018
GO:0006882	cellular zinc ion homeostasis	12	2	0.21	0.018
GO:0048812	neuron projection morphogenesis	183	5	3.22	0.018
GO:0046514	ceramide catabolic process	13	2	0.23	0.021
GO:0034312	diol biosynthetic process	13	2	0.23	0.021
GO:0007015	actin filament organization	213	7	3.75	0.025
GO:0043171	peptide catabolic process	15	2	0.26	0.028
GO:1901606	alpha-amino acid catabolic process	39	3	0.69	0.031
GO:0007200	phospholipase C-activating G protein-coupled receptor signaling pathway	39	3	0.69	0.031
GO:0002702	positive regulation of production of molecular mediator of immune response	16	2	0.28	0.032
GO:0006753	nucleoside phosphate metabolic process	238	4	4.19	0.035
GO:0031346	positive regulation of cell projection organization	38	3	0.67	0.035
GO:0019693	ribose phosphate metabolic process	172	2	3.03	0.035
GO:0006904	vesicle docking involved in exocytosis	17	2	0.3	0.035
GO:0006418	tRNA aminoacylation for protein translation	42	3	0.74	0.037
GO:0045944	positive regulation of transcription by RNA polymerase II	191	8	3.36	0.038
GO:0043473	pigmentation	18	2	0.32	0.039
GO:0018105	peptidyl-serine phosphorylation	74	4	1.3	0.041
GO:0035725	sodium ion transmembrane transport	78	4	1.37	0.049

Table S4. Summary of GO biological process enrichment analysis of the brown gene module identified by WGCNA.

GO ID	Term	Annotated	Significant	Expected	weightedFisher
GO:0006096	glycolytic process	27	9	0.48	5.40E-10
GO:0006457	protein folding	164	14	2.91	9.40E-07
GO:0030150	protein import into mitochondrial matrix	21	4	0.37	4.60E-04
GO:0032527	protein exit from endoplasmic reticulum	10	3	0.18	6.10E-04
GO:0031126	sno(s)RNA 3'-end processing	11	3	0.2	8.20E-04
GO:0006183	GTP biosynthetic process	11	3	0.2	8.20E-04
GO:0044772	mitotic cell cycle phase transition	99	7	1.76	0.001
GO:0009070	serine family amino acid biosynthetic process	13	3	0.23	0.001
GO:0007052	mitotic spindle organization	52	5	0.92	0.002
GO:0006767	water-soluble vitamin metabolic process	16	3	0.28	0.003
GO:0006094	gluconeogenesis	16	3	0.28	0.003
GO:0002183	cytoplasmic translational initiation	17	3	0.3	0.003
GO:0000413	protein peptidyl-prolyl isomerization	35	4	0.62	0.003
GO:0000079	regulation of cyclin-dependent protein serine/threonine kinase activity	36	4	0.64	0.004
GO:0006406	mRNA export from nucleus	35	4	0.62	0.006
GO:0015986	ATP synthesis coupled proton transport	21	3	0.37	0.006
GO:0030042	actin filament depolymerization	36	4	0.64	0.006
GO:0015931	nucleobase-containing compound transport	79	7	1.4	0.007
GO:0006913	nucleocytoplasmic transport	123	11	2.18	0.008
GO:1901264	carbohydrate derivative transport	24	3	0.43	0.009
GO:0006006	glucose metabolic process	63	7	1.12	0.009
GO:0042176	regulation of protein catabolic process	77	4	1.37	0.010

GO:0034314	Arp2/3 complex-mediated actin nucleation	25	3	0.44	0.010
GO:0006119	oxidative phosphorylation	66	4	1.17	0.010
GO:0006606	protein import into nucleus	61	5	1.08	0.012
GO:0009263	deoxyribonucleotide biosynthetic process	10	2	0.18	0.013
GO:0071456	cellular response to hypoxia	10	2	0.18	0.013
GO:0048026	positive regulation of mRNA splicing, via spliceosome	10	2	0.18	0.013
GO:0006415	translational termination	11	2	0.2	0.016
GO:0006544	glycine metabolic process	11	2	0.2	0.016
GO:0046031	ADP metabolic process	28	10	0.5	0.017
GO:0040011	locomotion	449	10	7.97	0.018
GO:0000075	cell cycle checkpoint signaling	60	3	1.07	0.018
GO:2001022	positive regulation of response to DNA damage stimulus	14	2	0.25	0.018
GO:0030834	regulation of actin filament depolymerization	29	2	0.52	0.018
GO:0006260	DNA replication	88	5	1.56	0.018
GO:0044282	small molecule catabolic process	148	4	2.63	0.019
GO:0046653	tetrahydrofolate metabolic process	13	2	0.23	0.022
GO:0000054	ribosomal subunit export from nucleus	13	2	0.23	0.022
GO:0072698	protein localization to microtubule cytoskeleton	13	2	0.23	0.022
GO:0043648	dicarboxylic acid metabolic process	34	3	0.6	0.022
GO:0043161	proteasome-mediated ubiquitin-dependent protein catabolic process	224	10	3.98	0.025
GO:0046847	filopodium assembly	15	2	0.27	0.028
GO:0006626	protein targeting to mitochondrion	38	6	0.67	0.035
GO:0034249	negative regulation of cellular amide metabolic process	70	3	1.24	0.035
GO:0090114	COPII-coated vesicle budding	17	2	0.3	0.036
GO:0051014	actin filament severing	17	2	0.3	0.036

GO:0008637	apoptotic mitochondrial changes	17	2	0.3	0.036
GO:0006122	mitochondrial electron transport, ubiquinol to cytochrome c	17	2	0.3	0.036
GO:0006418	tRNA aminoacylation for protein translation	42	3	0.75	0.038
GO:0044275	cellular carbohydrate catabolic process	18	2	0.32	0.040
GO:0009116	nucleoside metabolic process	19	2	0.34	0.044
GO:0007098	centrosome cycle	54	3	0.96	0.048
GO:0000278	mitotic cell cycle	321	21	5.7	0.049

Table S5. Summary of KEGG pathway enrichment analysis of the brown gene module identified by WGCNA.

ID	Description	GeneRatio	BgRatio	pvalue	qvalue
map05014	Amyotrophic lateral sclerosis	29/171	262/5881	2.65E-10	5.49E-08
map05012	Parkinson disease	24/171	192/5881	9.28E-10	5.49E-08
map03050	Proteasome	12/171	41/5881	9.43E-10	5.49E-08
map05020	Prion disease	24/171	193/5881	1.03E-09	5.49E-08
map05016	Huntington disease	23/171	211/5881	3.09E-08	1.15E-06
map00010	Glycolysis / Gluconeogenesis	10/171	35/5881	3.25E-08	1.15E-06
map01230	Biosynthesis of amino acids	10/171	45/5881	4.39E-07	1.33E-05
map05022	Pathways of neurodegeneration - multiple diseases	27/171	345/5881	1.76E-06	4.69E-05
map05010	Alzheimer disease	23/171	277/5881	4.18E-06	9.58E-05
map05017	Spinocerebellar ataxia	14/171	114/5881	4.51E-06	9.58E-05
map04066	HIF-1 signaling pathway	11/171	75/5881	8.87E-06	1.71E-04
map01110	Biosynthesis of secondary metabolites	23/171	294/5881	1.13E-05	2.01E-04
map01200	Carbon metabolism	11/171	80/5881	1.67E-05	2.74E-04
map00680	Methane metabolism	5/171	15/5881	4.65E-05	7.06E-04
map01120	Microbial metabolism in diverse environments	12/171	124/5881	2.33E-04	0.003
map05169	Epstein-Barr virus infection	13/171	145/5881	2.75E-04	0.004
map05415	Diabetic cardiomyopathy	13/171	146/5881	2.95E-04	0.004
map01232	Nucleotide metabolism	7/171	51/5881	6.11E-04	0.007
map05130	Pathogenic Escherichia coli infection	11/171	119/5881	6.31E-04	0.007
map00710	Carbon fixation by Calvin cycle	4/171	15/5881	7.33E-04	0.008
map00983	Drug metabolism - other enzymes	5/171	29/5881	0.001	0.013
map05132	Salmonella infection	13/171	173/5881	0.001	0.014
map04217	Necroptosis	9/171	94/5881	0.002	0.014
map05134	Legionellosis	6/171	44/5881	0.002	0.014
map00190	Oxidative phosphorylation	9/171	97/5881	0.002	0.016
map04115	p53 signaling pathway	7/171	66/5881	0.003	0.023
map04382	Cornified envelope formation	8/171	84/5881	0.003	0.023
map03013	Nucleocytoplasmic transport	8/171	87/5881	0.004	0.027
map04110	Cell cycle	10/171	129/5881	0.004	0.030

map04922	Glucagon signaling pathway	6/171	55/5881	0.005	0.035
map04218	Cellular senescence	9/171	113/5881	0.005	0.037

Table S6. Summary of GO biological process enrichment analysis of the turquoise gene module identified by WGCNA.

GO ID	Term	Annotated	Significant	Expected	weightedFisher
GO:0030198	extracellular matrix organization	75	17	2.48	2.60E-10
GO:0050919	negative chemotaxis	21	8	0.7	1.90E-07
GO:0007169	transmembrane receptor protein tyrosine kinase signaling pathway	223	29	7.39	4.10E-07
GO:0002040	sprouting angiogenesis	11	6	0.36	5.10E-07
GO:0001755	neural crest cell migration	25	8	0.83	9.00E-07
GO:0071526	semaphorin-plexin signaling pathway	28	7	0.93	2.70E-05
GO:0033674	positive regulation of kinase activity	88	12	2.92	3.20E-05
GO:0048843	negative regulation of axon extension involved in axon guidance	20	6	0.66	3.30E-05
GO:0060070	canonical Wnt signaling pathway	104	13	3.45	8.10E-05
GO:0030036	actin cytoskeleton organization	316	25	10.47	8.50E-05
GO:0034446	substrate adhesion-dependent cell spreading	15	5	0.5	8.90E-05
GO:0007275	multicellular organism development	1112	94	36.84	1.20E-04
GO:0030335	positive regulation of cell migration	70	11	2.32	1.30E-04
GO:0021782	glial cell development	10	4	0.33	2.10E-04
GO:0007399	nervous system development	546	45	18.09	3.10E-04
GO:0001936	regulation of endothelial cell proliferation	11	4	0.36	3.30E-04
GO:0050679	positive regulation of epithelial cell proliferation	11	4	0.36	3.30E-04
GO:0016477	cell migration	295	35	9.77	3.90E-04
GO:0042552	myelination	21	5	0.7	5.10E-04
GO:0030282	bone mineralization	13	4	0.43	6.70E-04
GO:0030178	negative regulation of Wnt signaling pathway	49	6	1.62	9.10E-04
GO:0007160	cell-matrix adhesion	49	7	1.62	0.001
GO:0048856	anatomical structure development	1359	110	45.02	0.001
GO:0010810	regulation of cell-substrate adhesion	15	4	0.5	0.001
GO:0007155	cell adhesion	456	46	15.11	0.002

GO:0035567	non-canonical Wnt signaling pathway	18	4	0.6	0.003
GO:0007156	homophilic cell adhesion via plasma membrane adhesion molecules	106	10	3.51	0.003
GO:0032963	collagen metabolic process	24	5	0.8	0.003
GO:0045766	positive regulation of angiogenesis	10	3	0.33	0.004
GO:0050918	positive chemotaxis	10	3	0.33	0.004
GO:0006468	protein phosphorylation	674	38	22.33	0.004
GO:0030204	chondroitin sulfate metabolic process	11	3	0.36	0.005
GO:0001944	vasculature development	73	14	2.42	0.006
GO:0043149	stress fiber assembly	12	3	0.4	0.006
GO:0007417	central nervous system development	113	10	3.74	0.006
GO:0072659	protein localization to plasma membrane	72	7	2.39	0.007
GO:0035023	regulation of Rho protein signal transduction	38	5	1.26	0.008
GO:0009790	embryo development	86	8	2.85	0.009
GO:0000122	negative regulation of transcription by RNA polymerase II	229	15	7.59	0.009
GO:0007411	axon guidance	93	13	3.08	0.010
GO:0007266	Rho protein signal transduction	52	8	1.72	0.010
GO:0043542	endothelial cell migration	14	3	0.46	0.010
GO:0097529	myeloid leukocyte migration	50	4	1.66	0.010
GO:0030154	cell differentiation	901	58	29.85	0.012
GO:0045892	negative regulation of transcription, DNA-templated	290	21	9.61	0.014
GO:0008360	regulation of cell shape	30	4	0.99	0.016
GO:0002690	positive regulation of leukocyte chemotaxis	17	3	0.56	0.017
GO:0006904	vesicle docking involved in exocytosis	17	3	0.56	0.017
GO:0007219	Notch signaling pathway	47	5	1.56	0.019
GO:0001525	angiogenesis	62	12	2.05	0.020
GO:0098742	cell-cell adhesion via plasma-membrane adhesion molecules	131	13	4.34	0.020

GO:0007517	muscle organ development	18	3	0.6	0.020
GO:0014706	striated muscle tissue development	20	3	0.66	0.021
GO:0043547	positive regulation of GTPase activity	100	7	3.31	0.022
GO:0017157	regulation of exocytosis	52	5	1.72	0.023
GO:0007179	transforming growth factor beta receptor signaling pathway	34	4	1.13	0.025
GO:0061564	axon development	153	16	5.07	0.026
GO:2000026	regulation of multicellular organismal development	155	19	5.13	0.026
GO:0052652	cyclic purine nucleotide metabolic process	22	4	0.73	0.027
GO:0090263	positive regulation of canonical Wnt signaling pathway	20	3	0.66	0.027
GO:0051016	barbed-end actin filament capping	20	3	0.66	0.027
GO:0008543	fibroblast growth factor receptor signaling pathway	35	4	1.16	0.028
GO:0006629	lipid metabolic process	632	15	20.94	0.028
GO:2000649	regulation of sodium ion transmembrane transporter activity	21	3	0.7	0.031
GO:0030574	collagen catabolic process	21	3	0.7	0.031
GO:0001666	response to hypoxia	21	3	0.7	0.031
GO:0050768	negative regulation of neurogenesis	22	7	0.73	0.033
GO:0032940	secretion by cell	171	11	5.66	0.033
GO:0061572	actin filament bundle organization	41	5	1.36	0.033
GO:0043086	negative regulation of catalytic activity	127	7	4.21	0.034
GO:0007165	signal transduction	2715	150	89.94	0.035
GO:0035329	hippo signaling	23	3	0.76	0.039
GO:0006906	vesicle fusion	57	5	1.89	0.040
GO:0030032	lamellipodium assembly	10	2	0.33	0.041
GO:0038084	vascular endothelial growth factor signaling pathway	10	2	0.33	0.041
GO:0120034	positive regulation of plasma membrane bounded cell projection assembly	10	2	0.33	0.041

GO:0048384	retinoic acid receptor signaling pathway	10	2	0.33	0.041
GO:0048009	insulin-like growth factor receptor signaling pathway	10	2	0.33	0.041
GO:0009065	glutamine family amino acid catabolic process	10	2	0.33	0.041
GO:0060395	SMAD protein signal transduction	40	4	1.33	0.042
GO:0007265	Ras protein signal transduction	144	16	4.77	0.043
GO:0016339	calcium-dependent cell-cell adhesion via plasma membrane cell adhesion molecules	24	3	0.8	0.044
GO:0006536	glutamate metabolic process	11	2	0.36	0.049
GO:0010632	regulation of epithelial cell migration	11	2	0.36	0.049

Table S7. Summary of GO biological process enrichment analysis of the purple gene module identified by WGCNA.

GO ID	Term	Annotated	Significant	Expected	weightedFisher
GO:0006482	protein demethylation	15	2	0.06	0.002
GO:0018107	peptidyl-threonine phosphorylation	18	2	0.07	0.002
GO:0018105	peptidyl-serine phosphorylation	74	3	0.28	0.003
GO:0035329	hippo signaling	23	2	0.09	0.004
GO:0000245	spliceosomal complex assembly	25	2	0.1	0.004
GO:1902115	regulation of organelle assembly	31	2	0.12	0.006
GO:0006357	regulation of transcription by RNA polymerase II	1371	13	5.27	0.009
GO:0030509	BMP signaling pathway	68	2	0.26	0.028
GO:0120034	positive regulation of plasma membrane bounded cell projection assembly	10	1	0.04	0.038
GO:0031114	regulation of microtubule depolymerization	10	1	0.04	0.038
GO:0018023	peptidyl-lysine trimethylation	10	1	0.04	0.038
GO:0032388	positive regulation of intracellular transport	11	1	0.04	0.042
GO:0045022	early endosome to late endosome transport	11	1	0.04	0.042
GO:0036010	protein localization to endosome	11	1	0.04	0.042
GO:0048638	regulation of developmental growth	36	2	0.14	0.044
GO:0048704	embryonic skeletal system morphogenesis	12	1	0.05	0.045
GO:1903829	positive regulation of cellular protein localization	12	1	0.05	0.045
GO:0007265	Ras protein signal transduction	144	3	0.55	0.047
GO:0072331	signal transduction by p53 class mediator	13	1	0.05	0.049
GO:0072698	protein localization to microtubule cytoskeleton	13	1	0.05	0.049
GO:0048268	clathrin coat assembly	13	1	0.05	0.049
GO:0034453	microtubule anchoring	13	1	0.05	0.049

Table S8. Summary of GO biological process enrichment analysis of the midnightblue gene module identified by WGCNA.

GO ID	Term	Annotated	Significant	Expected	weightedFisher
GO:0051607	defense response to virus	44	8	0.06	4.00E-16
GO:0009615	response to virus	47	10	0.06	1.70E-06
GO:0045071	negative regulation of viral genome replication	14	2	0.02	1.60E-04
GO:0031507	heterochromatin assembly	14	2	0.02	1.60E-04
GO:0045087	innate immune response	136	3	0.19	8.10E-04
GO:0001819	positive regulation of cytokine production	42	2	0.06	0.002
GO:0016553	base conversion or substitution editing	10	1	0.01	0.014
GO:0009263	deoxyribonucleotide biosynthetic process	10	1	0.01	0.014
GO:0009394	2'-deoxyribonucleotide metabolic process	11	1	0.02	0.015
GO:0042832	defense response to protozoan	13	1	0.02	0.018
GO:0009148	pyrimidine nucleoside triphosphate biosynthetic process	13	1	0.02	0.018
GO:0060968	regulation of gene silencing	14	1	0.02	0.019
GO:0051252	regulation of RNA metabolic process	2007	4	2.76	0.019
GO:0006221	pyrimidine nucleotide biosynthetic process	22	1	0.03	0.030
GO:0016925	protein sumoylation	31	1	0.04	0.042
GO:0034968	histone lysine methylation	32	1	0.04	0.043
GO:0050830	defense response to Gram-positive bacterium	33	1	0.05	0.045
GO:0007259	receptor signaling pathway via JAK-STAT	36	1	0.05	0.048
GO:0002376	immune system process	748	13	1.03	0.049

Table S9. Summary of KEGG pathway enrichment analysis of the turquoise gene module identified by WGCNA.

ID	Description	GeneRatio	BgRatio	pvalue	qvalue
map04510	Focal adhesion	29/262	145/5881	3.85E-12	7.99E-10
map05200	Pathways in cancer	46/262	395/5881	7.49E-10	7.77E-08
map04514	Cell adhesion molecules	21/262	106/5881	5.04E-09	3.48E-07
map04820	Cytoskeleton in muscle cells	25/262	153/5881	1.08E-08	5.62E-07
map04015	Rap1 signaling pathway	25/262	155/5881	1.42E-08	5.91E-07
map04151	PI3K-Akt signaling pathway	32/262	252/5881	5.12E-08	1.77E-06
map05205	Proteoglycans in cancer	22/262	158/5881	1.51E-06	4.48E-05
map04916	Melanogenesis	13/262	66/5881	4.97E-06	1.29E-04
map04014	Ras signaling pathway	22/262	174/5881	7.67E-06	1.77E-04
map04310	Wnt signaling pathway	17/262	119/5881	1.73E-05	3.59E-04
map05416	Viral myocarditis	10/262	45/5881	2.08E-05	3.91E-04
map04390	Hippo signaling pathway	16/262	113/5881	3.44E-05	5.94E-04
map04072	Phospholipase D signaling pathway	15/262	102/5881	3.85E-05	6.15E-04
map04360	Axon guidance	16/262	123/5881	9.82E-05	0.001
map04512	ECM-receptor interaction	11/262	64/5881	1.01E-04	0.001
map05412	Arrhythmogenic right ventricular cardiomyopathy	12/262	76/5881	1.15E-04	0.001
map05224	Breast cancer	14/262	100/5881	1.21E-04	0.001
map04926	Relaxin signaling pathway	13/262	89/5881	1.36E-04	0.002
map05146	Amoebiasis	11/262	67/5881	1.55E-04	0.002
map05226	Gastric cancer	14/262	103/5881	1.67E-04	0.002
map05414	Dilated cardiomyopathy	13/262	93/5881	2.15E-04	0.002
map05165	Human papillomavirus infection	22/262	218/5881	2.47E-04	0.002
map04010	MAPK signaling pathway	23/262	236/5881	2.99E-04	0.003
map04391	Hippo signaling pathway - fly	8/262	40/5881	3.09E-04	0.003
map04933	AGE-RAGE signaling pathway in diabetic complications	11/262	73/5881	3.38E-04	0.003
map05225	Hepatocellular carcinoma	14/262	111/5881	3.71E-04	0.003
map05410	Hypertrophic cardiomyopathy	12/262	87/5881	4.25E-04	0.003
map04020	Calcium signaling pathway	19/262	188/5881	6.45E-04	0.005
map05206	MicroRNAs in cancer	15/262	132/5881	7.15E-04	0.005
map04540	Gap junction	9/262	57/5881	8.27E-04	0.006

map04520	Adherens junction	11/262	81/5881	8.40E-04	0.006
map05218	Melanoma	8/262	48/5881	0.001	0.007
map04392	Hippo signaling pathway - multiple species	5/262	19/5881	0.001	0.007
map04928	Parathyroid hormone synthesis, secretion and action	11/262	85/5881	0.001	0.008
map04925	Aldosterone synthesis and secretion	9/262	64/5881	0.002	0.011
map05144	Malaria	7/262	41/5881	0.002	0.011
map04970	Salivary secretion	8/262	53/5881	0.002	0.012
map04934	Cushing syndrome	13/262	120/5881	0.002	0.014
map04270	Vascular smooth muscle contraction	11/262	93/5881	0.003	0.014
map04964	Proximal tubule bicarbonate reclamation	4/262	14/5881	0.003	0.014
map04810	Regulation of actin cytoskeleton	16/262	168/5881	0.003	0.016
map05217	Basal cell carcinoma	7/262	47/5881	0.004	0.022
map04725	Cholinergic synapse	10/262	87/5881	0.005	0.024
map04670	Leukocyte transendothelial migration	9/262	74/5881	0.005	0.025
map04929	GnRH secretion	7/262	49/5881	0.006	0.026
map04912	GnRH signaling pathway	8/262	62/5881	0.006	0.026
map04915	Estrogen signaling pathway	9/262	76/5881	0.006	0.027
map04022	cGMP-PKG signaling pathway	12/262	119/5881	0.006	0.027
map01521	EGFR tyrosine kinase inhibitor resistance	8/262	63/5881	0.006	0.027
map04935	Growth hormone synthesis, secretion and action	10/262	91/5881	0.007	0.029
map04911	Insulin secretion	8/262	64/5881	0.007	0.029
map04350	TGF-beta signaling pathway	10/262	94/5881	0.009	0.034
map04973	Carbohydrate digestion and absorption	4/262	19/5881	0.009	0.034
map04927	Cortisol synthesis and secretion	7/262	54/5881	0.009	0.036

Table S10. Summary of KEGG pathway enrichment analysis of the midnightblue gene module identified by WGCNA.

ID	Description	GeneRatio	BgRatio	pvalue	qvalue
map05164	Influenza A	8/15	110/5881	6.69E-11	1.27E-09
map04622	RIG-I-like receptor signaling pathway	6/15	52/5881	1.67E-09	1.59E-08
map05162	Measles	7/15	107/5881	3.09E-09	1.95E-08
map05160	Hepatitis C	6/15	100/5881	9.19E-08	4.35E-07
map05171	Coronavirus disease - COVID-19	6/15	185/5881	3.53E-06	1.34E-05
map05168	Herpes simplex virus 1 infection	5/15	126/5881	1.05E-05	3.33E-05
map05161	Hepatitis B	4/15	122/5881	2.02E-04	5.46E-04
map05169	Epstein-Barr virus infection	4/15	145/5881	3.92E-04	8.86E-04
map04623	Cytosolic DNA-sensing pathway	3/15	60/5881	4.21E-04	8.86E-04
map04621	NOD-like receptor signaling pathway	3/15	126/5881	0.004	0.007
map04620	Toll-like receptor signaling pathway	2/15	77/5881	0.016	0.026
map05165	Human papillomavirus infection	3/15	218/5881	0.016	0.026
